# The Antidepressant Drug Clomipramine Inhibits the ABC Transporter BmrA

**DOI:** 10.1002/cbic.202500937

**Published:** 2026-06-07

**Authors:** Nadja Hellmann, Christian Kersten, Thomas Efferth, Dirk Schneider

**Affiliations:** ^1^ Department of Chemistry ‐ Biochemistry Johannes Gutenberg University Mainz Germany; ^2^ Institute of Pharmaceutical and Biomedical Sciences ‐ Medical Chemistry Johannes Gutenberg‐University Mainz Germany; ^3^ Institute for Quantitative and Computational Biosciences Johannes Gutenberg‐University Mainz Germany; ^4^ Institute of Pharmaceutical and Biomedical Sciences ‐ Pharmaceutical Biology Johannes Gutenberg University Mainz Germany; ^5^ Institute of Molecular Physiology Johannes Gutenberg University Mainz Germany

**Keywords:** ABC transporter, BmrA, clomipramine, inhibitor, multidrug resistance

## Abstract

Drug uptake is constrained by epithelial barriers, such as the intestine and the blood–brain barrier, which express efflux pumps such as the ABC‐transporter ABCB1. Therefore, drugs targeting the central nervous system frequently require co‐administration of efflux inhibitors to facilitate sufficient target engagement. Conversely, certain drugs designed to target distinct molecular pathways may inadvertently affect ABC transporters. However, detailed functional studies of putative human ABCB1 inhibitors is challenging due to difficulties in obtaining sufficiently high amounts of protein needed. Bacterial ABC transporters, such as BmrA from *Bacillus subtilis*, share structural and functional similarities with ABCB1 and thus provide a reasonable alternative model system. This study investigates clomipramine, an antidepressant known to reduce multidrug resistance in human cells by a yet undefined mechanism. We demonstrate that clomipramine inhibits the ATPase activity of both full‐length BmrA and its isolated nucleotide‐binding domain. Using binding assays and molecular modeling, multiple clomipramine binding sites were identified in BmrA, which are also conserved in human ABCB1. Critically, one predicted site overlaps with the ATP‐binding pocket. The results underscore the potential side‐effects of clomipramine and highlight the value of bacterial models such as BmrA for studying novel inhibitors when studies on human proteins are not feasible.

## Graphical abstract

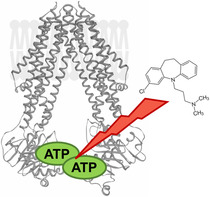

## 1 Introduction

ATP‐binding cassette (ABC) transporters utilize the energy gained via ATP hydrolysis to actively transport a large variety of substrates across cellular membranes. These transporters are present in all domains of life and share a common architecture [1]. The canonical structure of an ABC transporter encompasses two intertwined transmembrane domains (TMDs) with the substrate binding site, plus two cytosolic nucleotide‐binding domains (NBDs), which bind and hydrolyze ATP. There are different construction principles, yet two major variants to reach the canonical ABC transporter architecture: in eukaryotic ABC transporters, typically all domains are present on a single polypeptide chain, while in bacterial variants often two half‐transporters, each containing one TMD and one NBD, homo‐, or hetero‐dimerize [2, 3]. In either case, ATP hydrolysis is coupled to TM substrate transport via conformational changes. In fact, ABC‐transporters can adopt a whole range of conformational sub‐states, depending on the transport cycle and also on the substrate bound [4, 5, 6]. However, the ATPase activity always requires dimerization of the NBDs, since both protomers contribute to the ATP binding site. This ATP‐induced dimerization, together with structural changes induced by substrate binding, triggers conformational changes in the TMDs, finally allowing the substrate to exit [7, 8]. The transmission interface between the TMDs and the NBDs, coupling ATP hydrolysis to transport, is formed between residues around the Q‐loop of the NBDs and so‐called coupling helices (CHs) of the TMDs [3, 9, 10].

ABC transporters are classified based on their typical structural properties [3, 11]. Type IV ABC transporters are the largest group, which includes both dimeric and monomeric variants, where all the domains are part of a single polypeptide chain. In the dimeric representatives of this ABC transporter class, the coupling helix CH1 connects the NBDs and the TMDs of the same monomer, whereas CH2 connects the NBDs to the TMDs of the adjacent monomer [12].

ABC transporters are often involved in multidrug resistance. The human ABC transporter P‐glycoprotein (ABCB1), also known as MDR1, is expressed in a number of organs, e.g., liver, kidney, and brain. It is also found at the blood‐brain barrier (BBB) and limits the accumulation of xenobiotics in the brain. This is considered to be one of the causes of reduced effectiveness of therapies for neurodegenerative diseases or mental disorders [13, 14].

For finding inhibitors, a potential starting point is virtual screening using compound libraries as the first step, and candidate substances then have to be tested in functional assays [15, 16]. Often, these tests are performed in cell cultures employing a fluorescent substrate. In these assays, reduced or absent substrate transport indicates transporter inhibition. Generally, inhibitors that bind to substrate binding sites can be divided into two classes: (i) those, which are in fact a substrate, and therefore compete with the physiological substrates for transport, and (ii) non‐substrate inhibitors, which could bind either competitively or non‐competitively. Inhibitors that are not substrates are considered to be more promising drug candidates, since they are not eliminated by the transporter [17]. In recent years, the NBDs of ABC transporters are considered potential targets for inhibiting transport by eliminating the ATPase activity [18, 19].

Revealing the exact mode of action of a putative ABCB1 inhibitor requires controlled in vitro experiments. Yet, expression and purification of ABCB1 in sufficient amounts for performing functional in vitro analyses are challenging. Thus, using a related ABC transporter, which is easier to produce in reasonably high amounts, as a model system might better enable first screening approaches and subsequent detailed in vitro studies. The ABC transporter LmrA from *Lactococcus lactis* has emerged as such an ABCB1 replacement [20], as the transporter has been shown to be able to replace human ABCB1, at least in cell culture experiments [21]. In recent years, the LmrA homolog BmrA, an ABC transporter of *Bacillus subtilis*, is often used as a model ABC transporter. Like ABCB1, BmrA belongs to the class IV ABC transporter family, and thus, the transporters share a common topology. The ABC transporters ABCB1 and BmrA have similar substrate‐binding properties and share a number of substrates [22]. Via heterologous expression in *E. coli*, BmrA can be obtained in reasonably large amounts, which enables in vitro analyses [23, 24, 25]. Furthermore, employing inverted membrane vesicles from *E. coli* cells expressing BmrA, the transport activity can easily be tested, which can well replace costly cell‐culture experiments in the first‐line testing of compounds.

Here, we analyzed the interaction of clomipramine with BmrA as a proof of principle. Clomipramine was introduced as a drug for treating psychic disorders and is FDA approved for the treatment of obsessive–compulsive disorders [26]. A number of off‐label applications is listed, such as treatment of depression or pain [27]. Clomipramine, like other antidepressants, acts via binding to the serotonin receptor (SERT) [28]. However, it is also able to reverse multidrug resistance when co‐applied with other substances [29, 30, 31, 32], indicating a possible interaction with ABCB1. Yet, clomipramine´s exact in vivo mode of action has not been clarified yet, but at present, it is considered to be an inhibitor rather than a substrate [33]. As thus far no information about any putative interaction sites is available, we now employed BmrA as model for ABCB1 to shed light on the mechanism of ABC transporter inhibition by clomipramine. Our analyses clearly revealed that clomipramine affects the BmrA function via inhibiting its ATPase activity. Four potential clomipramine binding sites were identified. Three of these involved solely interactions with the NBDs, with one overlapping with the ATP binding site. The fourth one is present solely in the full‐length protein, at the binding groove where CH1 connects to the NBDs. Importantly, the identified binding sites are conserved also in human ABCB1. Thus, while there might exist specific binding sites in defined ABC transporters (here BmrA), binding of clomipramine to the structurally conserved ATP (or nucleotide) binding sites of ABC transporters can generally be expected, ranking clomipramine as a general ABC transporter inhibitor.

## 2 Results

### 2.1 Clomipramine Inhibits the BmrA Transport Activity

**FIGURE 1:**
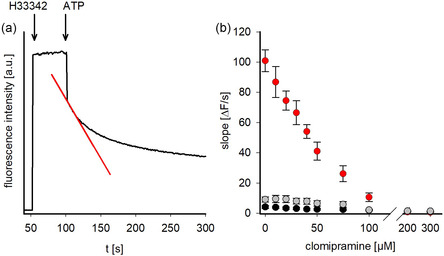
Inhibition of H33342 transport by clomipramine. (a) Raw data of a typical transport activity experiment. Upon addition of the fluorescent substrate (H33342) to inverted membrane vesicles, its fluorescence strongly increases due to incorporation of the dye into the membrane. After the addition of MgATP, H33342 transport by BmrA was initiated, leading to the removal of the dye from the membrane. As a measure for the transport activity, the initial slope (red line) was used. (b) Negative of the initial slopes measured at different clomipramine concentrations using inverted membrane vesicles containing wt BmrA (red), no BmrA (black), or inactive BmrA (mutant K308A, gray). Half‐maximal inhibition was observed at ≈44 µM clomipramine. *N* = 3 individual vesicle preparations; error bars indicate the SEM.

Inverted inner membrane vesicles prepared from BmrA‐expressing *E. coli* cells were isolated and incubated with 2 µM Hoechst33342 (H33342), a known BmrA (and ABCB1) substrate [22]. H33342 partitions into the vesicular membrane, accompanied by an increase in fluorescence. Addition of MgATP activates BmrA's transport activity, resulting in removal of membrane‐incorporated H33342, visible as a fluorescence decrease until a steady state is reached (Figure 1a). The negative slope of this decrease is a measure of BmrA's transport activity. To test whether clomipramine inhibits H33342 transport, inverted membrane vesicles were incubated with increasing concentrations of this compound prior to the addition of MgATP. As can be seen in Figure 1b, the BmrA activity decreases nearly linearly with increasing clomipramine concentrations until at around 100 µM clomipramine barely any activity remained. Thus, clomipramine clearly affects the amount of H33342 removed from the membrane by BmrA. As a control, the same experiment was performed with inverted membrane vesicles prepared from *E. coli* cells not expressing BmrA or harboring an inactive mutant (K308A) [34]. Here, the H33342 transport activity in the absence of clomipramine was already strongly reduced compared with the BmrA wt activity, as expected. Thus, the combined data clearly indicate inhibition of BmrA's H33342 transport activity by clomipramine. The concentration required to obtain half‐maximal inhibition in inverted membrane vesicles was about 44 µM.

### 2.2 Clomipramine Inhibits the BmrA ATPase Activity by a Mixed Mechanism

**FIGURE 2:**
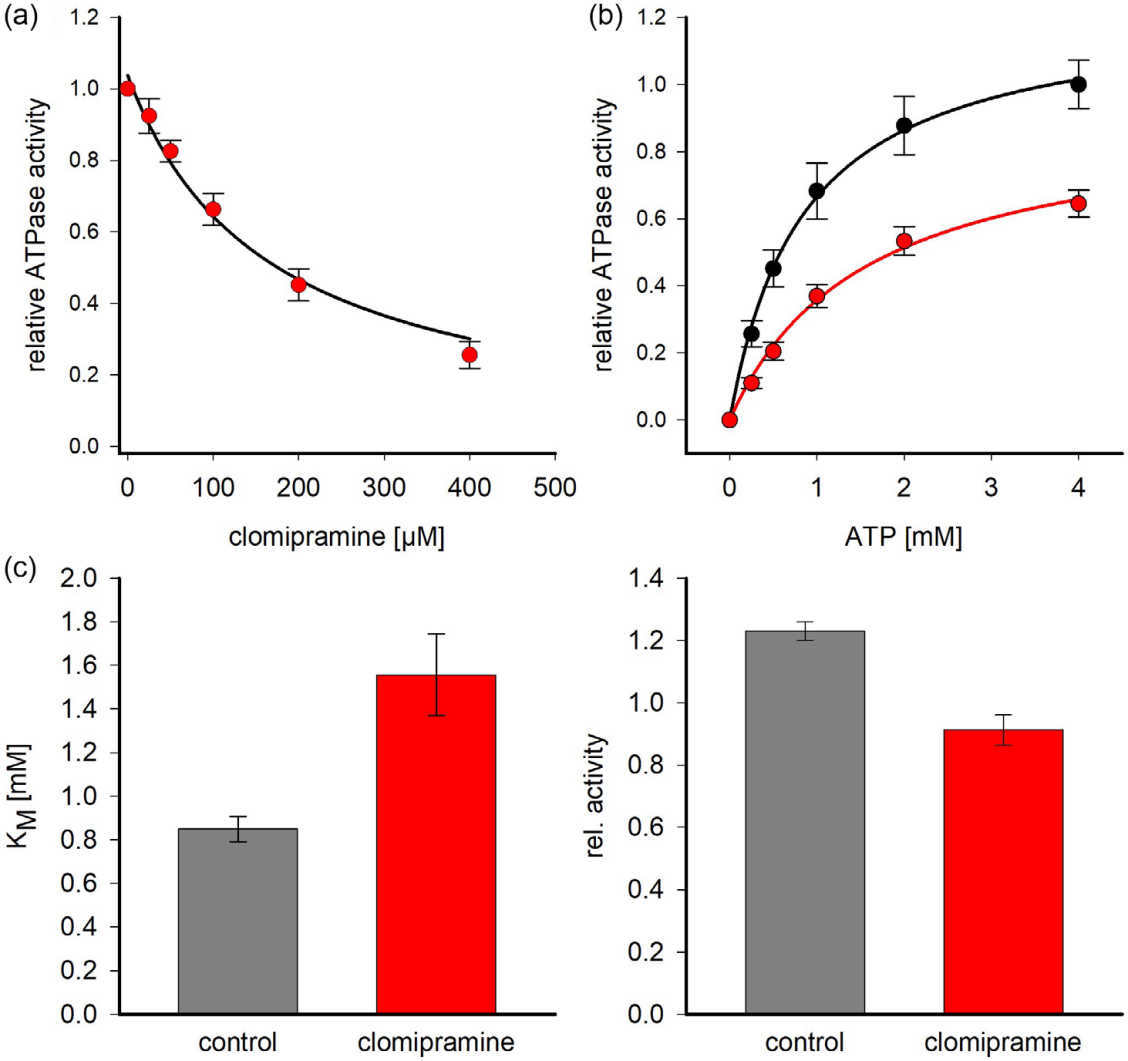
Clomipramine inhibits the ATPase activity of detergent‐solubilized BmrA. (a) ATPase activity of full‐length wt BmrA measured in detergent, normalized to the activity in the absence of clomipramine. The line corresponds to a fit based on a hyperbolic inhibition curve, yielding an IC_50_ of 163 ± 88 µM (Equation (1a)). *N* = 4–5, error bars represent the SEM. (b) ATPase activity of full‐length wt BmrA in detergent measured at different ATP concentrations in the absence or presence of 100 µM clomipramine. The activity was normalized to the activity measured in the control with 4 mM ATP. The lines correspond to a fit based on the Michaelis‐Menten equation (Equation (1b)). *N* = 3 technical replicates; error bars represent the SEM. (c) K_M_ values and the relative activities obtained from the fit in (b). The error bars represent the uncertainty in the fitted parameters estimated by the fitting routine. The actual k_cat_ of the control roughly varied between 50 and 100/min in the different preparations. Of note: The ATPase activity of BmrA in DDM micelles was inhibited by 80%–90% in the presence of 1000 µM orthovanadate, consistent with a prior report demonstrating comparable inhibition of BmrA in DDM at 500 µM orthovanadate [35].

When clomipramine was added to isolated BmrA wt in detergent, a clear reduction of the ATPase activity was observed, in line with the inhibited BmrA transport activity observed in inner membrane vesicles (Figure 2b). The curve could reasonably well be fitted with a hyperbolic function (eq. 1a), yielding an apparent IC_50_ of 163 ± 88 µM. The difference in the concentration of clomipramine required to obtain half‐maximal ATPase activity inhibition of the isolated protein in detergent micelles versus substrate transport mediated by the protein incorporated into membrane vesicles (Figure 1) could be caused by the different environment of BmrA (biological membrane vs. detergent micelle). However, when purified BmrA was reconstituted into EPL liposomes (Figure S1), its ATPase activity decreased with increasing clomipramine concentrations to a similar extent as observed for BmrA in detergent micelles, indicating that clomipramine interacts with BmrA in a comparable manner in DDM micelles and in an EPL bilayer. Possibly, the protein environment exerts a significant influence on the impact clomipramine has on BmrA‐mediated H33342 transport when assessed in inverted membrane vesicles.

Next, the dependence of the ATPase activity on the ATP concentration was measured in the absence *vs*. presence of clomipramine, to eventually reveal the mechanism of inhibition. The data obtained could reasonably well be fitted with a Michaelis‐Menten equation (Figure 2b, Equation (1b)). The presence of 100 µM clomipramine resulted in a decreased relative ATPase activity (v_rel_) yet an increased apparent K_M_ (Figure 2c). Consequently, inhibition of the BmrA ATPase activity by clomipramine cannot be explained by a single, simple inhibitory mechanism. While the increased K_M_ suggests inhibition due to competition with ATP binding, the decreased v_rel_ value indicates a more indirect, allosteric mechanism. In fact, the presence of (at least) two clomipramine binding sites is supported by a corresponding analysis of the transport data shown in Figure 1. Interestingly, in case of transport the sites seem to be coupled, as indicated by the strong cooperativity observed (Figure S2). To potentially disentangle the effects of clomipramine on the ATPase versus transport activity, the interaction of clomipramine with the isolated NBDs was next investigated.

### 2.3 The Isolated NBD of BmrA Binds Clomipramine

**FIGURE 3:**
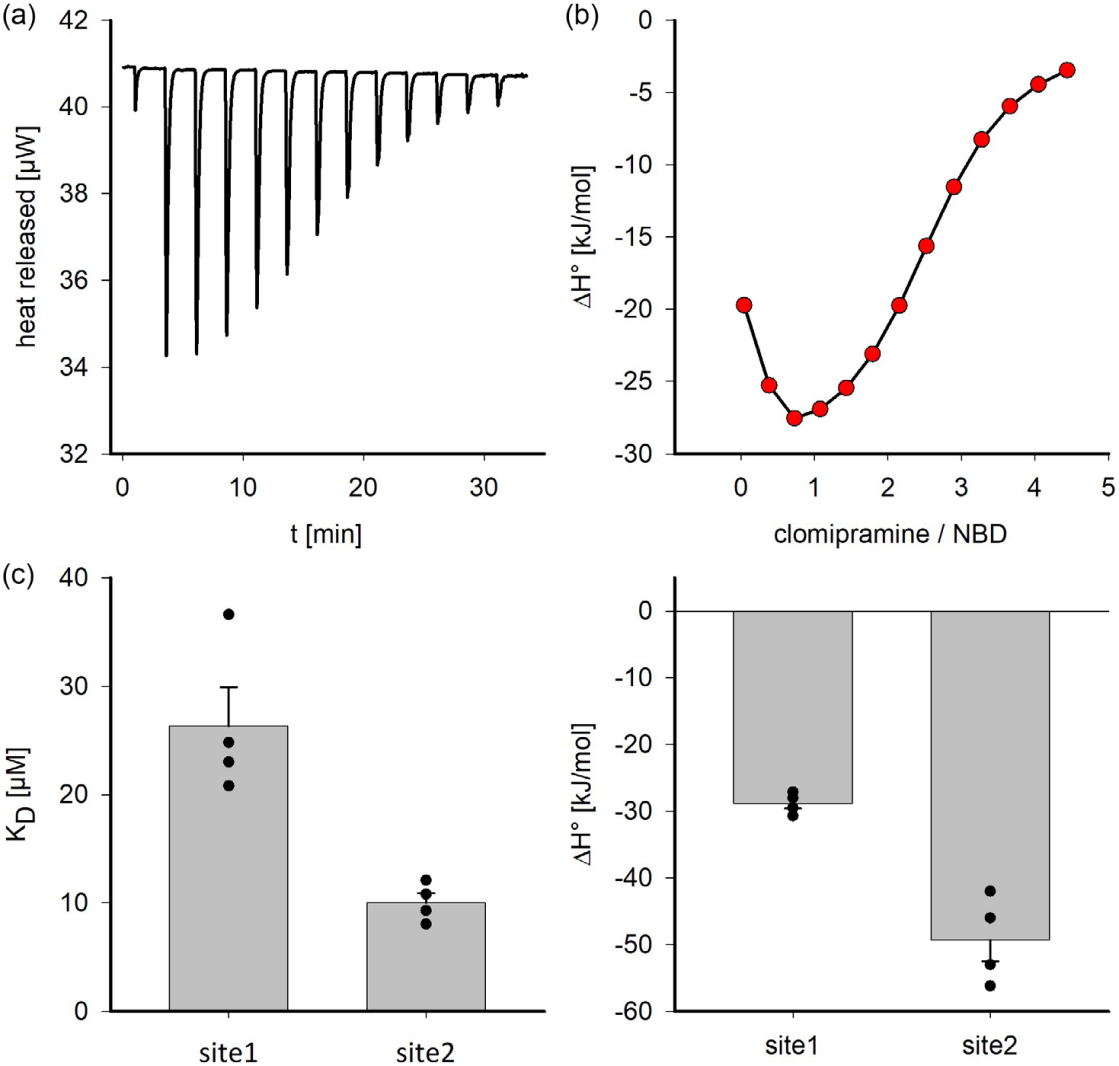
NBD offers two binding sites for clomipramine. Binding of clomipramine to the isolated BmrA NBD was investigated via Isothermal Titration Calorimetry in the absence of ATP. (a) One example of the titration of 1.2 mM clomipramine into 45 µM NBD, resulting in well‐shaped peaks. (b) The corresponding differential binding curve obtained from integration of the peaks indicates that a simple binding model is not applicable due to the hook‐like shape at the beginning of the titration. (c) Analysis of the binding curve based on a sequential binding model, assuming two binding sites per NBD, indicated positive cooperativity due to the higher K_D_ observed for site 1. Two replicates each of two individual protein preparations; the error bars reflect the SEM of the parameter values individually obtained from the four data sets.

Binding of clomipramine to the isolated BmrA wt NBD under equilibrium conditions was first investigated via Isothermal Titration Calorimetry (ITC), which allows determining the binding stoichiometry and binding affinities (Figure 3).

In the simplest case, with *n* identical binding sites per monomer, a sigmoidal shape of the ITC binding curve would be expected. Clearly, and consistently in all four performed independent measurements, the experimentally obtained curves deviated from such an ideal behavior, showing a “hook” at low molar ratios, often an indication of cooperative binding phenomena. Indeed, a model assuming sequential binding of clomipramine to two binding sites well described the data. The resulting K_D_‐values were *K*_D,1_ = 26.3 ± 3.6 and *K*_D,2_ = 10.0 ± 0.9 µM, indicating slightly positive cooperativity between the two binding sites (*K*_D,2_ < *K*_D,1_). While the “hook” could as well indicate the formation of BmrA NBD dimers induced by clomipramine binding, fitting the data and incorporating dimer formation would require a fitting equation including additionally clomipramine‐dependent dimerization, which contains too many parameters to be useful. Therefore, we next tested via crosslinking experiments whether NBD dimers form in solution, in the presence of clomipramine.

**FIGURE 4:**
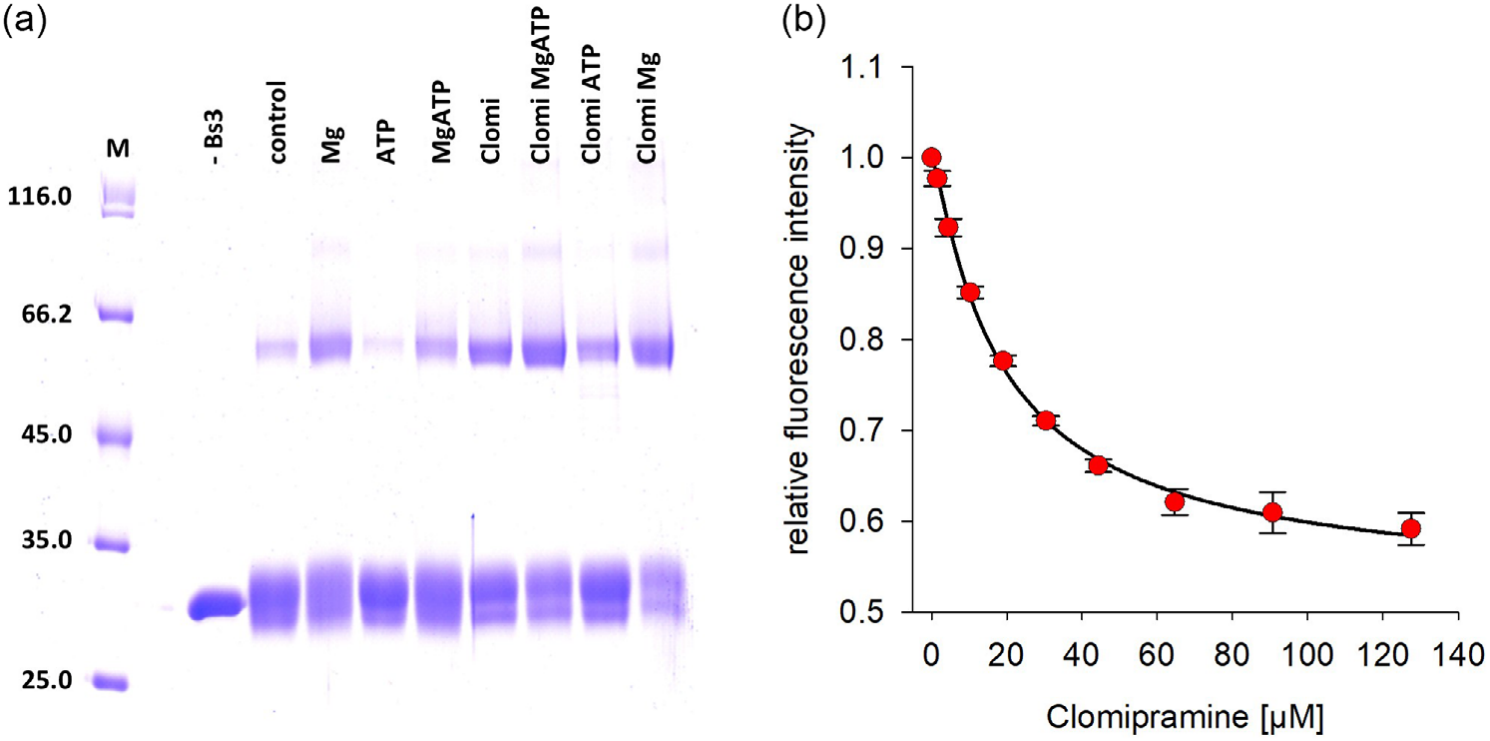
Clomipramine binding to the NBD and NBD dimer formation. (a) Isolated BmrA wt NBD at a concentration of 80 µM was incubated with or without different ligands, as indicated, prior to the addition of the crosslinker BS^3^. Clearly, the isolated NBD forms dimers in solution, and the presence of clomipramine increased the level of dimers in all cases. (b) The intrinsic fluorescence of the isolated monomeric BmrA wt NBD (1 µM) decreased by about 40% upon clomipramine addition (two technical replicates of two individual protein preparations; error bars reflect the SEM).

When isolated NBDs were incubated with the cross‐linker BS^3^, formation of NBD dimers was observed via SDS PAGE analyses, yet the dimer fractions were quite small (Figure 4a, control). The presence of solely Mg^2+^, as well as of Na_2_ATP plus Mg^2+^, increased the fraction of dimeric NBD. However, at each condition, the dimer level clearly was higher in the presence of clomipramine. Thus, clomipramine appears to stabilize a dimeric NBD assembly. However, clomipramine binding to the NBDs does not require dimerization per se*.* This was shown via monitoring binding of clomipramine to the NBDs by intrinsic fluorescence spectroscopy. Here, only a low protein concentration (1 µM) was employed. The cross‐linking experiments showed that even at 80 µM the fraction of dimerized NBDs was not very high and therefore is insignificant at 1 µM due to the quadratic dependence of dimer formation on the free protein concentration. Thus, at an NBD concentration of 1 µM, no significant changes in the Trp‐fluorescence would be expected if clomipramine exclusively binds to NBD dimers. Yet, the intrinsic protein fluorescence decreased by about 40% with increasing clomipramine concentrations (Figure 4b). Besides demonstrating that clomipramine binds (also) to monomeric NBDs, these results indicate that binding to at least one of the two potential binding sites affects the environment of the sole Trp in the NBD (W413). The *K*_D_‐value obtained via fitting a simple binding curve (Equation (4)) to the fluorescence data (Figure 4b) was 24 +/− 6 µM, in very good agreement with one of the K_D_‐values obtained in the ITC‐experiments (Figure 3).

### 2.4 The Isolated NBD of BmrA Is Catalytically Active and Inhibited by Clomipramine

To study the impact clomipramine has on the isolated NBDs in more detail, the impact of the compound on the ATPase activity of isolated NBDs was next analyzed.

**FIGURE 5:**
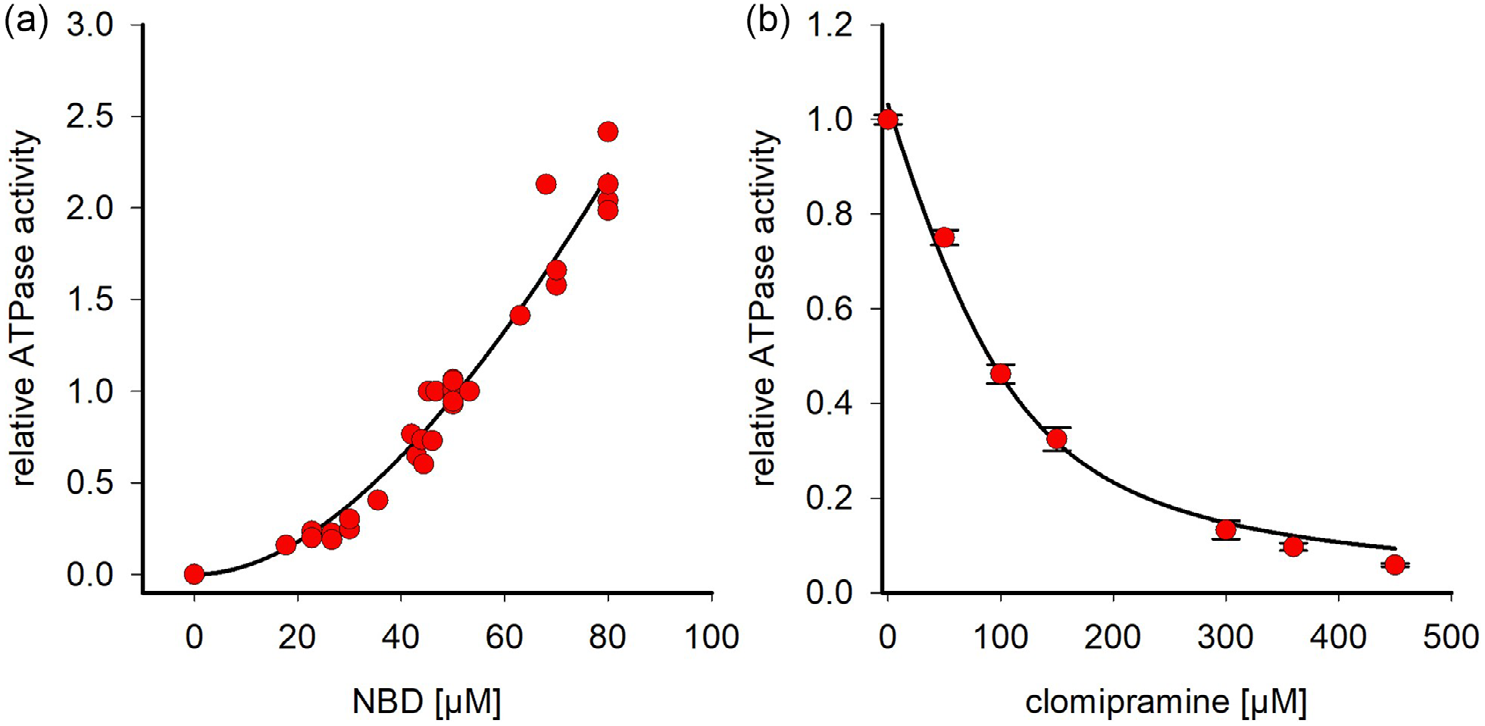
The isolated NBD is an active ATPase and inhibited by clomipramine. (a) The isolated NBD of BmrA wt forms ATPase active dimers. Data obtained from seven different experiments (five different preparations) are combined, after normalizing the values from each set to the value obtained at 50 µM NBD. This was necessary due to variations in the absolute activity of the different samples. The activity increases roughly quadratically with protein concentration, indicating dimer formation. The activity of 50 µM NBD was about 0.033/min. The line presents the fit based on Equation (2). (b) The ATPase activity of 50 µM isolated NBD was measured at different clomipramine concentrations and normalized to the corresponding control. The normalized values were averaged (*N* = 3 with two individual protein preparations; error bars indicate the SEM). The line represents a fit based on Equation (3), considering the reduction of available clomipramine due to binding to two sites of the NBD.

Full‐length BmrA is a constitutive dimer in detergent, held together by interactions within the TMDs [35]. For ATP hydrolysis, ATP has to be sandwiched between two NBDs; thus, the ATPase activity requires formation of NBD dimers [8]. Consequently, isolated NBDs hydrolyze ATP only if they are able to form dimers in solution. When the ATPase activity of the isolated NBDs was monitored at very high protein concentrations, clearly an ATPase activity was detected (Figure 5a). Furthermore, the nonlinear dependence of the observed ATPase activity on the protein concentration reflects the requirement of dimer formation. Thus, dimerization of the NBDs is indeed responsible for the activity, and not any potentially co‐purified impurity. In fact, as can be seen in Figure S3, the protein was highly pure, and no contaminations were observed even when extremely high protein concentrations were loaded on a gel.

By fitting an appropriate dimer model to the data (Equation (2)), an apparent dimerization constant of 600 +/− 300 µM was estimated, predicting that 10%–20% monomers were present as dimers at 50 µM [NBD]. The average apparent *k*_cat_ of a solution containing 50 µM NBD was about 0.033/min. However, to obtain a proper *k*_cat_ value, the fraction of dimers has to be considered. Thus, since only 10%–20% of all BmrA was dimeric, the real *k*_cat_ is 10–5 times higher than the actually measured value, yielding a *k*_cat_ value of 0.165–0.33/min. This can be regarded as a lower limit, since the measurements were not performed at ATP concentrations near *v*_max_ conditions. Of note, all our attempts to determine the *K*_M_ value failed due to protein aggregation at increasing Mg^2+^ concentrations. Thus, the fraction of the NBD loaded with ATP could not be properly estimated.

Nevertheless, the NBD's *k*_cat_ value clearly is well below the value determined for the full‐length protein in detergent (50–100/min, Figure 2). Yet, the activity was sufficiently high to allow investigating the impact clomipramine has on the activity of isolated NBD dimers. As can be seen in Figure 5b, the ATPase activity of the isolated NBDs steadily decreased with increasing clomipramine concentrations. A visual estimation of the IC_50_ yielded a value of about 100 µM. However, in contrast to the experiments with the full‐length BmrA protein, the protein concentration was much higher here, in the range of the inhibitor concentration, so that the actual inhibition constant K_I_ is much lower than the IC_50_. This is because a considerable amount of clomipramine was bound to the NBD, and thus the free drug concentration was unknown. Consequently, as a better approximation for the effective K_I_, Equation (3) was fitted to the data, yielding *K*_I_ = 54 ± 12 µM. Unfortunately, a rigorous, model‐based analysis was not possible, due to the large amounts of different species to be considered. This leads to a large number of unknown binding constants, which cannot be disentangled.

### 2.5 Predicted Druggable Sites on BmrA

To identify putative clomipramine binding sites, we next searched for druggable sites on the BmrA NBDs with *SiteFinder*. With this method, potential binding sites for compounds are predicted based on the chemical composition and structure at a protein's surface, on the basis of a training data set that contained the most accurate and diverse complex structures from the PDB [36]. Different structures of the NBD were investigated: the NBDs cut out from the closed (dimeric NBD) and the open (monomeric NBD) structures of full‐length BmrA (6R72 and 8REZ), respectively, plus a homology model of the NBD based on the structure of the NBD from MsbA (5IDV, Figure S4). The latter structure of the isolated NBD differs to some extent from the structure of the NBD in the context of the full‐length proteins (C_α_‐RMSD: 2.58 Å compared with 6R72 and 2.75 Å compared with 8REZ), and thus this structure potentially better reflects the structure of the isolated NBD analyzed in our experiments. Also, binding of clomipramine to the NBD in the context of the full‐length open structure (8REZ) was investigated. For each type of structure, multiple druggable sites were identified. In the following, the ones with the highest druggability score are discussed.

**FIGURE 6:**
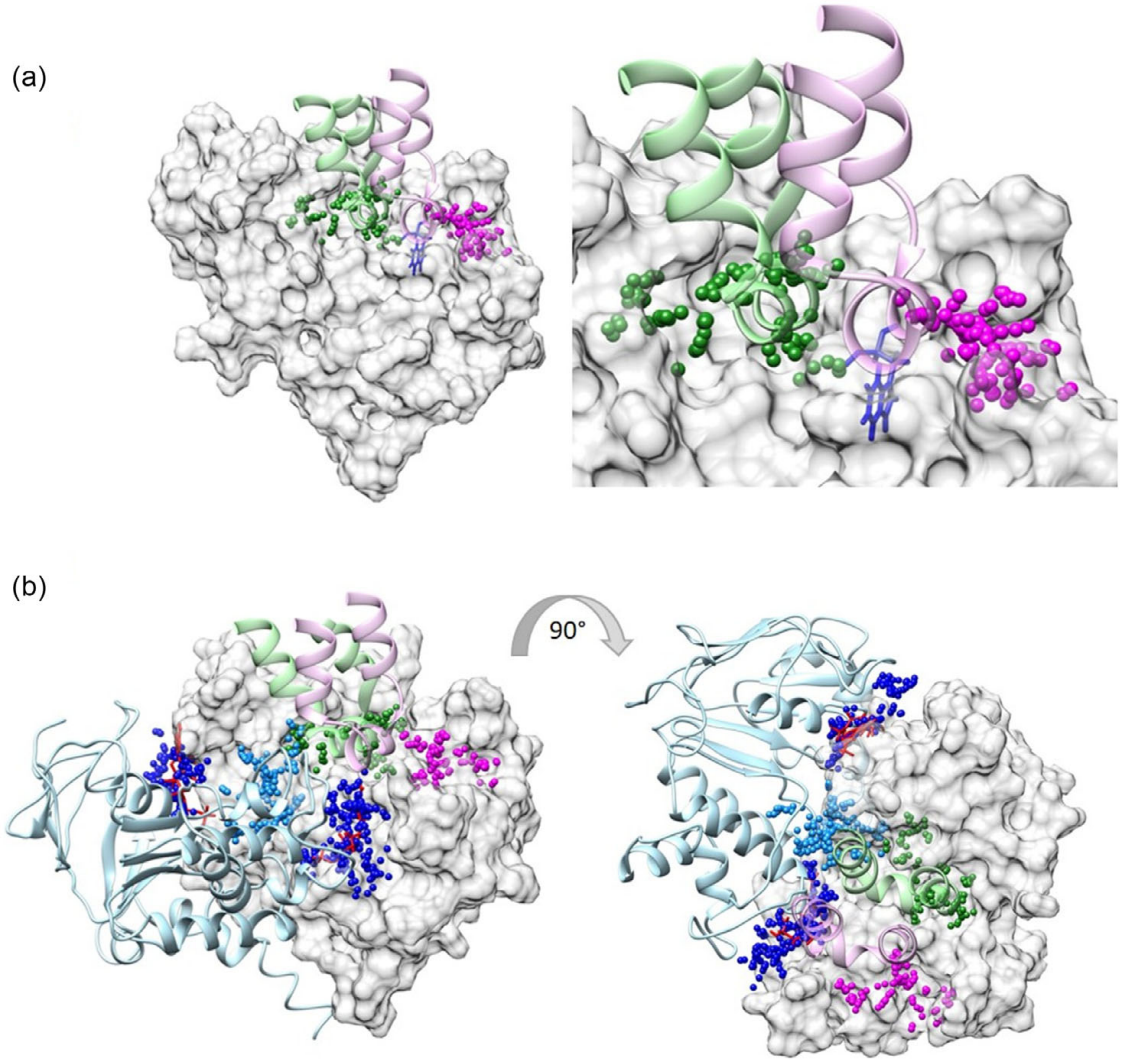
Potential clomipramine binding sites on the BmrA NBD. (a) Potential clomipramine interaction sites on the NBD monomer extracted from the structure of full‐length BmrA in the open conformation (6REZ). The NBD is shown as gray surface model. The binding site suggested for the isolated monomer is shown as green spheres. In the context of the full‐length protein, this binding site would lead to clashes with the CH2 from the second monomer (light green ribbon). The binding site suggested when the NBD is part of the full‐length structure (shown as magenta spheres) is located near the binding groove of CH1 (pink ribbons). The location of the sole Trp in the NBD is shown in blue. (b) Potential clomipramine binding sites identified when the NBDs are dimeric, i.e., when the full‐length BmrA is in the closed state (6R72). One NBD is shown as gray surface model, and the other as blue ribbon. In the dimeric structure, the sites with the highest druggability score were all located between the two protomers. Two (identical) clomipramine binding sites are suggested to overlap with the ATP binding sites (dark blue spheres), and an additional one is found in the center (light blue spheres). The bound ATP molecules are shown as red sticks.

Using the structure of full‐length BmrA in an open conformation (8REZ) as a template, we next searched for potential clomipramine binding sites in the NBDs, using on the one hand, the isolated monomer, cut out from 8REZ.pdb, and on the other hand the NBD in the context of the full‐length BmrA structure. For the isolated monomer, the preferred binding site indicated by the green spheres in Figure 6a was predicted. The same region was identified when the monomer derived from a homology model (5IDV, Figure S4) was used, demonstrating that the NBD structure in the context of the full‐length protein does not differ significantly from the isolated NBD structure. Yet, in the context of the full‐length protein, the predicted clomipramine binding site would lead to clashes with the CH2 from the second monomer (Figure 6a, light green ribbon). Consequently, this binding site was not identified when the full‐length BmrA structure was analyzed. However, a different binding site (magenta spheres) near the binding groove of CH1 (pink ribbons) was suggested. This binding region involves interactions between clomipramine and CH1 (see Table S1), which explains why clomipramine binding was not identified when the isolated NBD was analyzed. Of note, both binding sites are located near the sole Trp of BmrA's NBD. Additional potential clomipramine binding sites were identified when the dimeric NBDs, cut out from the full‐length structure in the closed state (6R72), were analyzed. One binding site was suggested to overlap with the ATP binding sites (Figure 6b, dark blue spheres, one per monomer), and an additional one in the center between two interacting monomers (light blue spheres). The identified binding region located at the ATP binding site was also identified when full‐length human ABCB1 was analyzed (Figure S5a) albeit the overall region considered as potential binding sites is larger. Similarly, for the NBDs in the full‐length open structure (8GMG) or the isolated NBD cut out from this structure, the regions defined as binding site are more extended, but the location is similar to the one identified on BmrA's NBDs (Figure S5b,c). Also, some of the suggested binding sites on the isolated ABCB1 monomer are not found in the full‐length structure. Thus, different clomipramine binding sites potentially exist on the NBD, which are, however, not all accessible in the full‐length context.

## 3 Discussion

The antidepressant drug clomipramine is considered to be a non‐substrate inhibitor of human ABCB1, yet its model of action still is enigmatic. The aim of the present study was to clarify how clomipramine inhibits an ABC transporter. As a manageable model, the bacterial ABCB1 homolog BmrA was analyzed, which shares structural and functional properties with human ABCB1.

Since the solubility of clomipramine was not sufficiently high at pH 8.0, all experiments were performed at pH 7.0 and at 25°C, in contrast to other studies described in the literature. Nevertheless, the *k*_cat_ values for the ATPase activity of full‐length BmrA in DDM obtained in this study are in a similar range as previously described: For example, in [35], the activity at 37°C and pH 8.0 in DDM was about 80 min^−1^, compared with 50–100 min^−1^ in our study (Figure 2). Thus, BmrA can be considered highly active also in the now used somewhat different buffer conditions. Furthermore, we could now show that the isolated BmrA NBD is able to dimerize in solution and hydrolyzes ATP at sufficiently high protein concentrations (Figure 5). The hydrolysis rate k_cat_ of the dimeric NBD is considerably lower than the value obtained with the full‐length protein (Figure 3). Based on our analysis of the dependence of the observed rate on protein concentration, we estimate that approximately 10%–20% of the monomers exist in a dimeric form at 50 µM [NBD]. This low level of dimers is in agreement with the cross‐linking experiments (Figure 4a). So far, based on NMR measurements, the isolated NBD of BmrA was considered to be unable to form dimers [37]. Yet, in these experiments, low levels of dimers might be difficult to detect.

### 3.1 Clomipramine Inhibits the BmrA ATPase Activity

Overall, the results presented here unambiguously show that clomipramine binds to BmrA and functionally affects its ATPase as well as substrate transport activity. The ATPase activity of the full‐length protein as well as of the isolated NBD was strongly reduced in the presence of clomipramine. Consequently, also the BmrA transport activity determined in inverted membrane vesicles was affected, as ATP hydrolysis fuels the transmembrane substrate transport. The inhibition constant derived from the decrease in BmrA's ATPase activity in the isolated state was in the low tens of µM, in agreement with the K_D_‐value determined based on ITC and fluorescence binding studies (Figure 3, 4). However, our data cannot be explained by assuming a simple inhibition mechanism, since clomipramine likely binds to different BmrA regions, resulting in site‐specific effects.

### 3.2 Multiple Clomipramine Binding Sites on BmrA

For the simplest system, the isolated, monomeric NBD, our fluorescence emission measurements (Figure 4B) indicated a binding site in close proximity to the sole Trp413, in agreement with a binding region suggested by *SiteFinder*, which actually included Trp413 as a potentially interacting residue (Table S1). Binding to this site could well affect ATP binding, since it is suggested to involve the amino acids Arg484, Ile487, Ala488, Arg491, and Arg495, (Table S1), which are located right next to the ABC signature sequence (^479^LeuSerGlyGlyGln^483^). This ABC signature sequence is part of the ATP binding site formed by two interacting NBDs. In fact, addition of ATP reduced clomipramine binding to the monomeric NBD at the site near Trp413 (Figure S6). This shows that ATP binds to the monomeric NBD and impacts clomipramine binding, but obviously without directly affecting the environment of Trp413, since no changed fluorescence was observed in the presence of solely ATP (Figure S6). Furthermore, it was recently suggested that a three‐residue cluster with Trp413 in the center senses nucleotide binding [9]. However, the clomipramine binding site suggested for the isolated monomeric NBD with the highest score (Figure 6a) is probably not relevant for the inhibition of the ATPase activity in the full‐length protein context, where this site is occupied by CH2. For the full‐length BmrA in the open conformation, clomipramine binding at another region, namely near the CH1 binding groove, was predicted by *SiteFinder* (Figure 6b). Of note, this site was not predicted in the isolated NBD, probably since CH1 also forms contacts with clomipramine (see Table S1). Likely, the interactions of clomipramine with solely residues of the NBD, i.e., in the absence of the stabilizing CH1 contacts, are not sufficiently strong to be detected as a potential binding site. Noteworthy, clomipramine binding to this region would lead to some clashes with CH1 when BmrA is in the closed conformation (Figure S4). Thus, binding to this site might stabilize the open BmrA conformation, thereby affecting the BmrA ATPase cycle, in line with our results.

In line with the crosslinking results obtained with the isolated NBD (Figure 4a), *SiteFinder* also suggested binding of clomipramine between the NBDs, at the ATP‐sites, when the NBDs are dimerized in the context of the full‐length BmrA dimer in its closed conformation (Figure 6b). Thus, competitive inhibition in the dimeric state is also likely.

The ITC binding experiments suggest the presence of two binding sites on an isolated monomer, which exhibit slight positive cooperativity (Figure 3). Noteworthy, based on our analysis (Figure 5) the NBD is essentially monomeric at the used protein concentrations. Of the possible clomipramine binding sites being discussed above, the most likely candidate for one of the sites identified via ITC is the site clashing with CH2 (green spheres, Figure 6a). The location nearby NBD's sole Trp supports this hypothesis. The second binding site is probably rather the ATP‐binding site than the site near the CH1 groove, since the latter has probably a low affinity in the absence of CH1. Furthermore, the impact ATP addition has on clomipramine binding (Figure S6) supports this hypothesis. Thus, it seems likely that inhibition of the NBD's ATPase activity is largely caused by binding of clomipramine to the monomer, thereby interfering with ATP binding. The slight cooperativity observed in the ITC experiments suggests that binding at the ATP site and the site near the CH2 groove are weakly coupled through conformational changes. When considering inhibition of the full‐length BmrA ATPase activity, the data overall suggest that competitive binding of clomipramine to the ATP‐binding site is part of the inhibitory mechanism, plus possibly (additionally) clomipramine binding to the CH1‐groove, disturbing the conformational transitions necessary for ATP hydrolysis. This would well explain the apparent mixed inhibition pattern observed in the steady state kinetics (Figure 2). Given the pronounced steepness of the transport–inhibition curve (Figure 1), we hypothesize that clomipramine binding to the CH1‐groove site impairs transport to a greater extent than ATPase activity, indicating a functional decoupling of the two processes. While the clomipramine binding sites identified in this study are all located on the NBDs, clomipramine binding to regions outside the NBDs, such as the TMDs, cannot be finally excluded, with potential further effects on the transport activity. In fact, for the thioxanthene cis‐(Z)‐fluxpentixol, which is structurally related to clomipramine, binding to ABCB1 at a site distinct from the substrate binding site, but at the TMDs, was postulated [38]. Unfortunately, our attempt to obtain more detailed information about the binding sites using electron microscopy failed, since BmrA precipitated at the protein concentrations required for structural analyzes, when clomipramine was present.

The affinity of clomipramine binding to the NBDs found in the present study is in the range of a few tens of micromolar, which is similar to what has been observed as typical concentrations to, e.g., inhibit dynamins GTPase activities, or a Ca^2+^‐ATPase [39, 40]. Nevertheless, reported plasma concentrations of clomipramine are typically below ≈1 µM [41, 42, 43]. Consequently, the IC_50_ values obtained in this study indicate that clomipramine is unlikely to be clinically relevant as a broad‐spectrum ATPase inhibitor. Moreover, owing to its partial lipophilicity, the apparent IC_50_ in human cell models is expected to be even higher, because the larger membrane‐to‐transporter ratio in cells reduces the effective drug concentration at the transporter. This now raises the question about the relevance of clomipramine as an inhibitor of ABC‐transporters in humans. While the evidence in experimental human models currently is limited, clomipramine has already been shown to inhibit ABCB1 in three canine cell lines [30].

While selectively targeting the ATP‐binding sites of the NBDs with an inhibitor is generally unsuitable, owing to the risk of broadly affecting cellular ATPases, the CH1 groove emerges as a promising pharmacological target [44]. This pocket is expected to confer greater specificity than the highly conserved ATP‐binding site and may also be more selective than the substrate‐binding site, given the polyspecific nature of BmrA, ABCB1 and other ABC transporters. Indeed, several ABC‐transporter inhibitors have been suggested to bind within the CH1 groove [44, 45].

Also in case of clomipramine, binding sites suggested by *SiteFinder* include regions on the NBDs which are located near the interface to the coupling helices, both for BmrA and ABCB1. However, given the diversity of potential binding sites for clomipramine identified by *SiteFinder* across different BmrA and ABCB1 structures, no single, well–defined interaction interface appears to be consistently favored. Nevertheless, using clomipramine as a structural scaffold, rational modifications could be designed to enhance binding specificity toward the target groove.

Studies such as the present one, employing BmrA as a model for in vitro screening and analyses, to identify possibly existing different modes of action, have the potential to foster identifying new site‐specific inhibitors in the near future.

## 4 Experimental Section

### 4.1 Expression and Purification of Full‐Length BmrA and the Isolated NBD

After heterologous expression in *E. coli* BL21(DE3)/pLysE (Merck KGaA, Darmstadt, GER), full‐length BmrA wt and variants were purified in the presence of detergent (DDM), and the soluble NBD variants without detergent, as described in detail before [23, 24]. Yet, due to the low solubility of clomipramine (Merck KGaA, Darmstadt, GER) at pH 8.0, we used HEPES‐KOH buffer at pH 7.0 instead of pH 8.0. Furthermore, the NBD was additionally purified via size exclusion chromatography (SEC) using a HiLoad 16/60 Superdex 200 column (Cytivia, Wilmington, USA), resulting in highly pure protein (Figure S3). Clomipramine stock solutions were prepared in HEPES‐KOH buffer, pH 7.0 at a concentration of 1.2 mM. For protein concentration determination via measuring the absorbance at 280 nm, the following calculated extinction coefficients (https://web.expasy.org/protparam/) were used: εBmrA‐wt = 38 850 M^−1^ cm^−1^ and εNBD = 15 930 M^−1^ cm^−1^.

Proteoliposomes were formed using *E. coli* polar lipids (EPL) from Avanti Polar Lipids (Birmingham, AL, USA) following the protocol described in (25), with the difference that HEPES‐KOH buffer at pH 7.0 was used instead of pH 8.0 for resuspension of the formed proteoliposomes.

### 4.2 BmrA Hoechst 33342 Transport Activity Measurements

BmrA transport activities were measured using inverted *E. coli* inner membrane vesicles and the dye Hoechst 33342 (2′‐[4‐ethoxyphenyl[‐5‐4‐] methyl‐1‐piperazinyl]−2,5′‐bis‐1H‐benzimidazole, Merck KGaA, Darmstadt, GER) as a substrate, as described before [24]. The activity was measured in 50 mM HEPES‐KOH, 2 mM MgCl_2_, 8.5 mM NaCl pH 7.0 buffer containing 4 mM phosphoenolpyruvate, and 20 µg/µL pyruvate kinase (from rabbit muscle, 350–600 units/mg, Merck KGaA, Darmstadt, Germany) as an ATP‐regenerating system. Inverted membrane vesicles with a protein concentration of 0.25 g/l were incubated in this buffer for 10 min at 25°C. The background fluorescence emission of this sample was measured for approximately 50 s (*λ*_ex_ = 355 nm, *λ*_em_ = 457 nm) with a FluoroMax‐4 fluorometer (Horiba Europe GmbH, Oberursel, Germany). Subsequently, Hoechst 33 342 was added to a final concentration of 2 µM, the sample was mixed, and the fluorescence was measured for another 50 s. Then, MgATP was added (final concentration of 2 mM) and the fluorescence was subsequently monitored for further ~200 s. The negative of the initial slope of the measured fluorescence intensity after ATP addition reflects the transport activity. As a control, inverted vesicles from *E. coli* cells not expressing BmrA or harboring an inactive variant (K308A) [34] were analyzed.

### 4.3 ATPase Activity Measurements

The ATPase activity of BmrA was measured with an ATP regenerating system, following the consumption of NADH via changes in its absorption at 340 nm using a Lambda 35 U V/Vis spectrophotometer (PerkinElmer, Inc., Waltham, MA, USA) for 10 min at 25°C. The solution contained Na_2_ATP, 8.5 mM MgCl_2_, 0.15 mM NADH, 2 mM phosphoenolpyruvate, and 2 µL of pyruvate kinase (600–1000 U/mL)/lactate dehydrogenase (900–1400 U/mL) mix (Merck KGaA, Darmstadt, Germany) in 200 µL of 50 mM HEPES‐KOH, pH 7.0. To convert the ADP formed during storage back to ATP, first, a mix of all components except BmrA was incubated for 5 min in 100 µL buffer, including clomipramine at the intended concentration. For the measurements, this sample was mixed with BmrA (0.2 µM in case of full‐length protein in DDM and 0.5 µM in case of proteoliposomes) in 100 µL buffer also containing the final clomipramine concentration. In the case of full‐length BmrA in DDM, the buffer contained additionally 5 mM DDM. The ATPase activity of the isolated NBD was measured at the indicated protein concentrations. The initial decline of the absorption at 340 nm was converted to a NADH consumption rate based on an extinction coefficient of 6220 M^−1^ cm^−1^.

The ATPase activities measured at different clomipramine concentrations [C] were normalized to the value determined in the absence of clomipramine for each measurement, averaged and analyzed based on a simple inhibition mechanism.

(1a) $$v_{rel}=a\left[1-\frac{\left[C\right]}{IC_{50}+\left[C\right]}\right]+b$$

The ATPase activity measured for the full‐length protein was normalized to the rate measured at 4 mM ATP for each preparation, and the mean of different preparations was calculated subsequently. The resulting curves were analyzed based on the Michaelis–Menten equation:

(1b) $$v_{rel}=v_{rel,max}\frac{\left[ATP\right]}{K_{M}+\left[ATP\right]}$$

The rates measured for the isolated NBDs obtained at different protein concentrations were normalized to the rate measured at 50 µM protein for each preparation, and the mean of different preparations was calculated subsequently. The resulting curve was analyzed based on a model considering a dimer/monomer equilibrium, and applying mass‐conservation with [NBD] referring to the total NBD concentration, to obtain

(2) $$v=v_{rel}\frac{1+4[NBD]/K_{dim}-\sqrt{1+8[\mathrm{NBD}]/K_{dim}})}{8}\;with\;v_{rel}=k_{cat}\theta_{A}K_{dim}$$

Here, the fraction of dimers occupied by ATP is given by *θ*_A_, which is unknown but constant in this experiment. The equilibrium constant *K*_dim_ relates the concentrations of dimers to monomers according to [all dimers] = [all monomers]^2^/*K*_dim_. “All dimers” and “all monomers” are the sum of concentrations of all dimers or monomers, with or without bound ATP.

The rates obtained for the NBD at 50 µM concentration at varying clomipramine concentrations were normalized to the rate determined in the complete absence of clomipramine for each preparation, and the mean was calculated subsequently. The curve obtained was analyzed based on a model describing the binding of clomipramine to two identical sites, and assuming that the clomipramine‐carrying NBDs cannot associate to a functional ATP‐bound dimer anymore. This yields the following fitting function:

(3) $$v=a\left[1-\frac{\left[C\right]+2\left[NBD\right]+K_{I}-\sqrt{{\left(\left[C\right]+2\left[NBD\right]+K_{I}\right)}^{2}-8\left[NBD\right]\left[C\right]}}{4\left[\mathrm{NBD}\right]}\right]$$

Here, *K*_I_ is an apparent inhibition constant, since no distinction is made for different types of binding (one or two clomipramine per monomer, or a combination of ATP and clomipramine bound). These possible states cannot be disentangled based on the performed experiments.

### 4.4 Binding of Clomipramine to the Isolated NBD

For the fluorescence‐based binding experiments, NBD in HEPES‐KOH, pH 7.0 at a concentration of 1 µM was incubated with clomipramine for 10 min at 25°C at the indicated concentrations. Trp fluorescence emission spectra of the proteins were measured with a FP 8500 fluorimeter (JASCO Deutschland GmbH, Pfungstadt, Germany), upon excitation of 295 nm. The intensities measured at 340 nm were normalized to the emission measured in the absence of clomipramine. To correct for possible inner filter effects due to absorption by clomipramine, the experiment was repeated with a free Trp solution (1 µM). The fluorescence of Trp decreased linearly to about 10% at 130 µM clomipramine. The relative intensity measured for the NBD was divided by the relative intensity of free Trp at the corresponding clomipramine concentration to correct for this effect. The resulting binding curve was analyzed based on a simple binding model:

(4) $$F_{norm}=1-a\frac{\left[C\right]}{K_{D}+[C]}$$

For the isothermal titration experiments (ITC), the MicroCal PEAQ‐ITC Automated system (Malvern Panalytical, Malvern UK) was used. A solution containing 1.2 mM clomipramine was titrated into a solution containing 45 µM NBD, both dissolved in HEPES‐KOH, pH 7.0. The titration involved 12 injections of 3 µL every 150 s at 25°C, except the first injection which was only 0.4 µL. As a control, the clomipramine solution was titrated with the same scheme into a cell exclusively containing the buffer, resulting in negligible peaks. Employing the software provided by the manufacturer, the peaks were integrated, the point for the first titration was removed, and the resulting binding curve was analyzed based on a model of “sequential binding” to two binding sites, as offered by the instrument's software package.

### 4.5 Crosslinking Experiments

To stabilize any formed dimers, 50 µL of 80 µM NBD in HEPES‐KOH, pH 7.0 were incubated for 15 min at room temperature with Na_2_ATP (3 mM), MgCl_2_ (8 mM), clomipramine (600 µM), or combinations thereof, as indicated. The crosslinker BS^3^ (bis(sulfosuccinimidyl) suberate, Thermo Fisher Scientific, Waltham, USA) was added to a final concentration of 1.6 mM, and the sample was incubated for 30 min at RT. To quench the reaction, 3 µL of a Tris/HCl solution (pH 7.6) was added to a final concentration of 50 mM, and the sample was incubated for 15 min. Then, the sample was mixed with SDS‐PAGE sample buffer, heated to 95°C, and analyzed via SDS‐PAGE. SDS‐PAGE gels were subsequentely stained with Coomassie brilliant blue R250.

### 4.6 Homology Modeling and Clomipramine Binding Site Predictions

Three‐dimensional structures of BrmA (PDB‐IDs 6R72, 8REZ) [46, 47]), of an *A. baumanii* MsbA NBD monomer (5IDV) [48], and of ABCB1 (PDB‐IDs 6C0V, 8GMG) were accessed via the protein data bank (PDB) [49, 50]. For druggability prediction of the BrmA NBD dimer in the closed conformation and the monomer cut out from the open conformation, the structures were truncated by removing residues prior Thr328. In case of ABCB1, the part between residues 380 and 630 was employed.

Structures were prepared using the QuickPrep functionality of MOE [51] to add missing atoms and for protonation. Subsequently, the *SiteFinder* [36] functionality was used for binding site and druggability predictions at default conditions. The approach employed by *SiteFinder* (“binding site probing”) is more suited to identify different types of sites than molecular docking. *SiteFinder* probes the protein surface and predicts possible ligand binding sites based on size and hydrophobicity matching and compares sites based on these properties. In contrast, coarse‐grained molecular docking compare sites on the basis of interaction properties and simplifies the effect of ligand binding on target dynamics and desolvation, which can be very different between different sites. Therefore, binding site probing is more reliable to find possible sites in general, but does not provide a predicted binding mode.

A BmrA NBD monomer homology model (residues 328–589) was built using the *A. baumanii* MsbA NBD as a template (5IDV). The sequence identity and similarity were 42% and 62%, respectively, as determined with MOE [51]. Ten initial structures were built and scored using the generalized born/volume integral (GB/VI) method [52]. The best scoring model showed no internal strain and clashes, and no outliers were found in the phi‐psi plot. It was subsequently subjected to *SiteFinder* as described above. For the human ABC transporter ABCB1, druggable binding sites were identified in a similar manner, based on the structure in the outward facing ABCB1 conformation (6C0V) and inward facing conformation (8GMG) [49, 50]. Here, the NBDs in the context of the full‐length protein, as well as isolated NBD monomers were investigated.

## Funding

This study was supported by the Deutsche Forschungsgemeinschaft (SFB1552 ‐ “Defects and Defect engineering in Soft Matter,” project number 465145163).

## Conflicts of Interest

The authors declare no conflicts of interest.

## Supporting information

Supplementary Material

## Data Availability

The data that support the findings of this study are available from the corresponding author upon reasonable request.
