# Supplementary material for: The Antidepressant Drug Clomipramine Inhibits the ABC Transporter BmrA
Source: Chembiochem. 2026 Jun 7;27(11):e202500937. doi: 10.1002/cbic.202500937 (PMC13243972; doi:10.1002/cbic.202500937)
Supplement: Supplementary file 1 — Supplementary Material [file CBIC-27-e202500937-s001.pdf]

Supporting Information

The antidepressant drug clomipramine inhibits the ABC transporter BmrA

Nadja Hellmann[a], Christian Kersten[b,c], Thomas Efferth[d], Dirk Schneider[a,e]

[a] Department of Chemistry - Biochemistry, Johannes Gutenberg University Mainz, Germany

[b] Institute of Pharmaceutical and Biomedical Sciences - Medical Chemistry, Johannes Gutenberg-University Mainz, Germany

[c] Institute for Quantitative and Computational Biosciences, Johannes Gutenberg-University Mainz, Germany

[d] Institute of Pharmaceutical and Biomedical Sciences - Pharmaceutical Biology, Johannes Gutenberg University Mainz, Germany

[e] Institute of Molecular Physiology, Johannes Gutenberg University Mainz, Germany

Figure S1: Inhibition of the full-length BmrA ATPase activity in proteoliposomes vs. detergent.

Figure S2. A model with two strongly interacting sites describes the transport inhibition curve.

Figure S3: Purity of the isolated BmrA NBD.

Figure S4. Clomipramine binding regions predicted on monomeric NBD structures.

Figure S5. Putative clomipramine binding sites identified at the ABCB1 NBD.

Figure S6. Reduction of clomipramine binding to BmrA's NBD upon addition of ATP.

Table S1. Residues suggested to be involved in clomipramine binding.

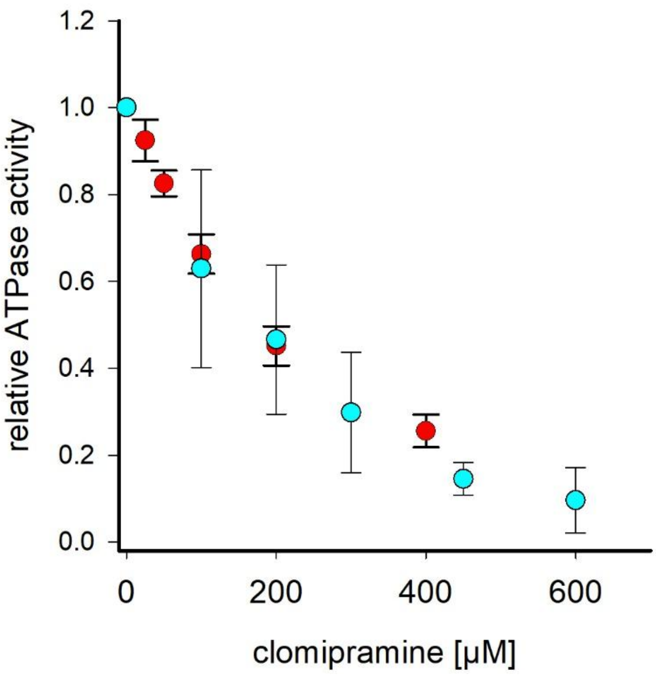

Figure S1. Inhibition of the full-length BmrA ATPase activity in proteoliposomes *vs.* detergent.

Inhibition of the ATPase activity was measured with full-length BmrA in micelles (red spheres: data from Figure 2 in main text) and reconstituted in EPL liposomes (cyan spheres). Both curves largely coincide, indicating that the interaction of clomipramine in DDM micelles and in an EPL bilayer is similar. The data with proteoliposomes are each the average of triplicates of data from four different BmrA preparations. Error bars represent SEM.

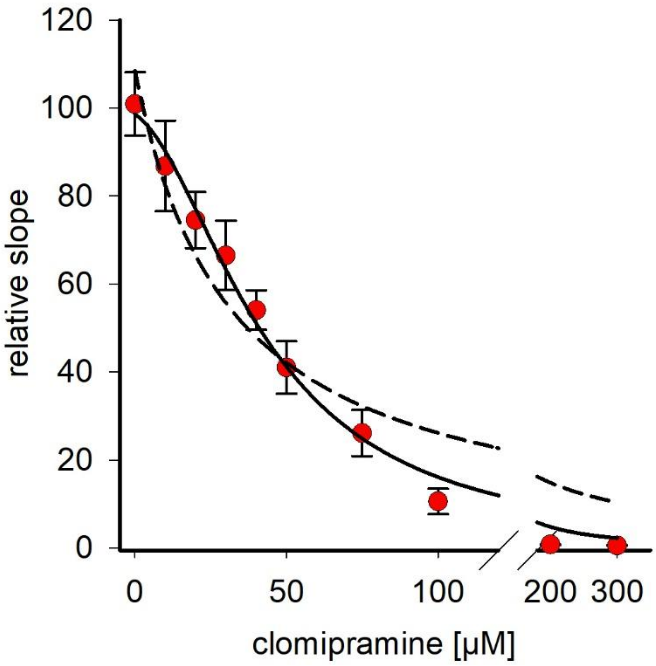

Figure S2. A model with two strongly interacting sites describes the transport inhibition curve.

The data shown in Figure 1 in the main text cannot be described by a simple inhibition curve ($v=v_o/(1+K*x)$, dashed line). However, allowing binding of two clomipramine molecules ($v=vo/(1+K_1*x+K_1*K_2*x^2)$) yields a much better fit (solid line). For K_1 a value of 0.0046 ± 0.0043 μM^{-1} was found; for K_2 a value of 0.10 ± 0.11 μM^{-1} .This suggests a strong cooperativity between the two binding sites.

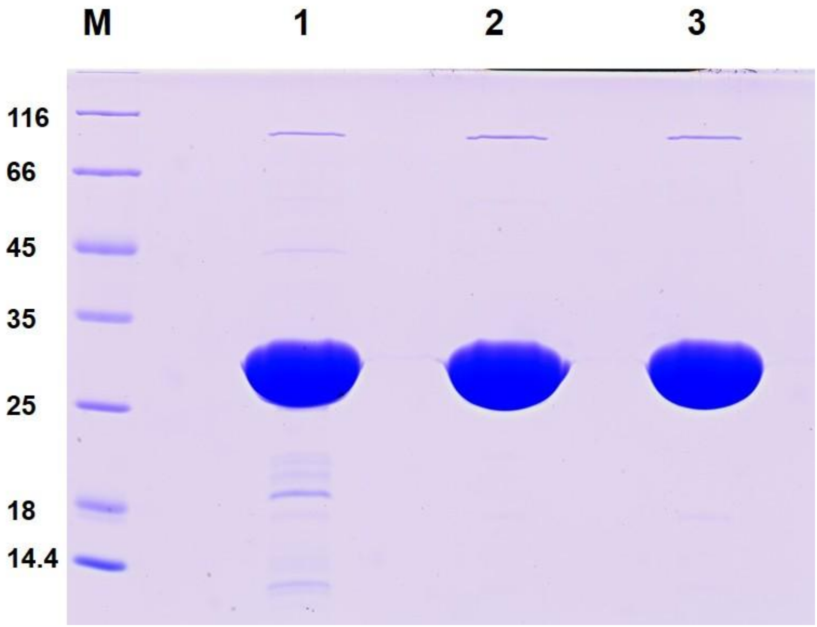

Figure S3. Purity of the isolated BmrA NBD.

The NBD isolated via Ni-NTA affinity chromatography was applied to a size exclusion column, and the three fractions of the main peak were analysed by SDS-PAGE. About 45 µg of protein was loaded in each lane. Fractions two and three were used for the described experiments. M: Molecular mass standards.

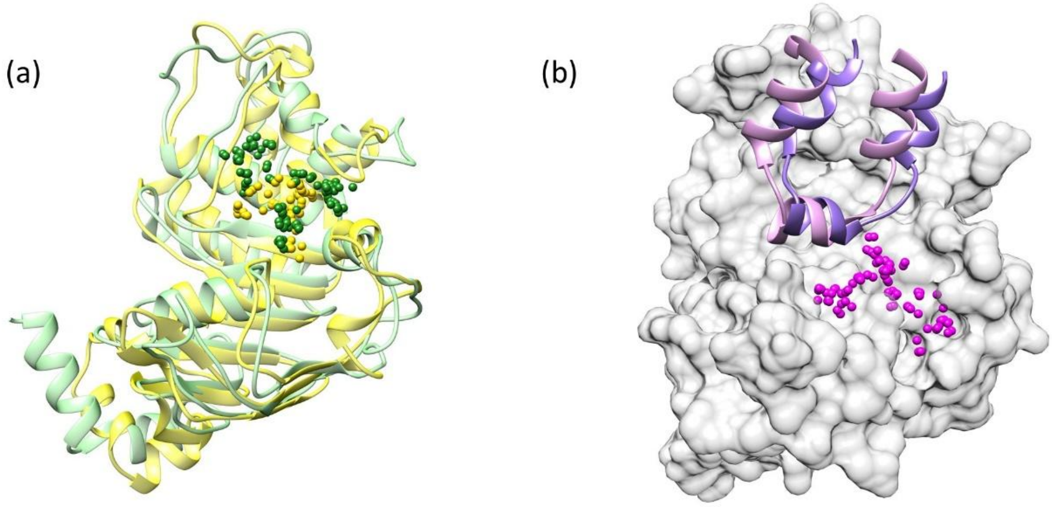

Figure S4. Clomipramine binding regions predicted on monomeric BmrA NBD structures.

a) Putative clomipramine binding sites identified by *SiteFinder* using the homology model of BmrA's NBD, based on the structure of the isolated NBD from MsbA (5IDV, yellow ribbon and yellow spheres) as a template. The structure is compared to the structure of the NBD monomer cut out from the open BmrA full-length structure (8REZ, green ribbons and green spheres). Despite slight differences in the overall structure, the putative binding sites are at very similar positions. b) Overlay of the binding sites suggested for the full-length protein in the open conformation (pink spheres, CH2 in pink ribbon) with the position of the CHs in the closed conformation (violet ribbon, 6R72). The structures were oriented to each other based on the NBDs. In the closed conformation, some residues of the putative clomipramine binding sites clash with CH2.

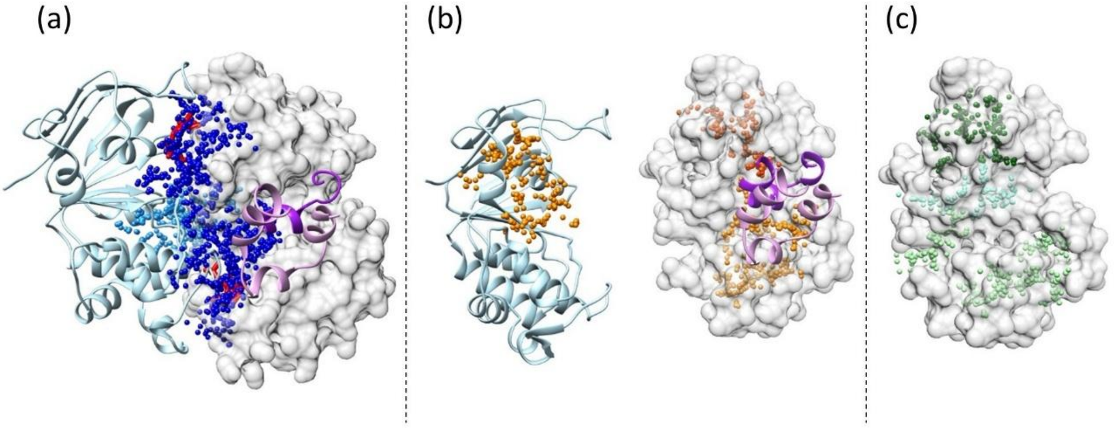

Figure S5. Putative clomipramine binding sites identified at the ABCB1 NBD.

a) Putative clomipramine binding sites predicted by *SiteFinder* for the dimeric NBDs cut out from the closed structure of human ABCB1 (PDB-Id. 6C0V). The bound ATP is shown in red, and the predicted clomipramine binding sites near the ATP site are indicated by dark blue spheres. Additional binding sites between the two NBDs are indicated by light blue spheres. One NBD is shown as grey surface model, the other one in light blue ribbons. For orientation, the coupling helices CH3 and CH4 are also shown as violet and light pink ribbons. b) The N-terminal NBD of ABCB1 in the open conformation (PDB-Id. 8GMG) was aligned to the same NBD in the closed structure. Putative clomipramine binding sites are depicted in shades of orange. Notably, the two NBDs appear to possess distinct regions exhibiting the highest binding scores. Again, the CHs are shown for orientation. c) Clomipramine binding sites as suggested by *SiteFinder* for the C-terminal NBD extracted from the open ABCB1 structure. The orientation is the same as in b); potential binding sites are shown by spheres in shades of green. Some but not all potential binding sites are occluded in the full-length structure.

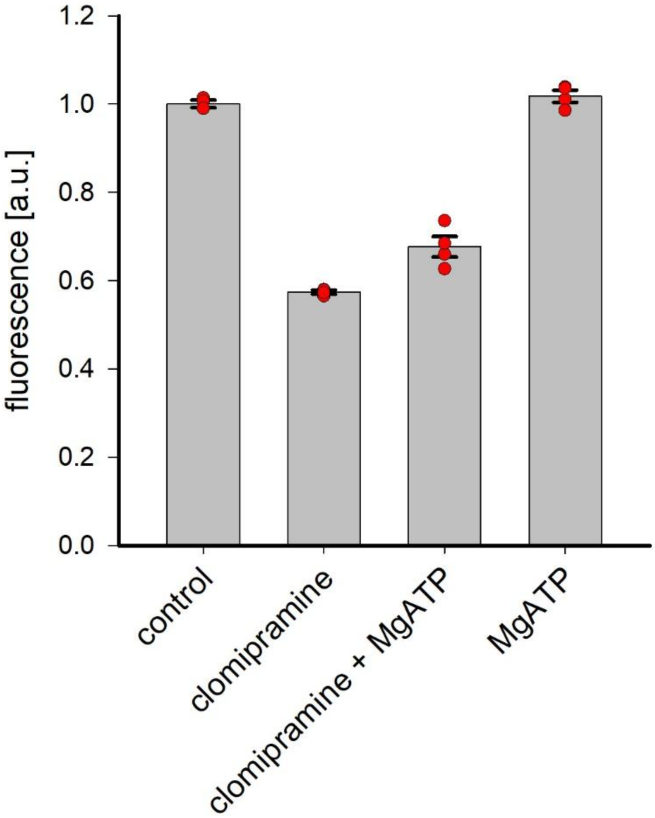

Figure S6. Reduced clomipramine binding to the BmrA NBD upon addition of ATP.

The decrease of the intrinsic Trp fluorescence was employed to monitor clomipramine binding, as shown in Figure 4b in the mani text. Upon addition of 100 µM clomipramine, the Trp fluorescence intensity dropped by 43%. In the presence of 3.5 mM ATP, 8.5 mM $MgCl_2$, the decrease was reduced, amounting to 32 %. Of note, binding of ATP itself did not lead to a decreased fluorescence intensity. N=3 technical replicates, the error reflects the standard error of the mean.

Table S1. Residues suggested to be involved in clomipramine binding by SiteFinder. Those, which are explicitly discussed in the main text, are highlighted in bold.

Structure	Site	Chain	Amino acids
**BmrA**			
Monomer model based on MsbA (5IDV)	1	-	Lys217 Lys385 Leu387 Glu388 Phe390 Trp413 Arg414 Ile417 Gly418 Tyr419 Glu423 Ser424 Pro425 Tyr437 Gly438 Glu440 Ala488 Arg491 Ala492 Arg495
Monomer extracted from the open conformation (8REZ)	1	-	Glu388 Phe390 Glu411 **Trp413** Arg414 Glu415 Ile417 Gly418 Tyr419 Val420 Ser424 Pro425 Leu426 Met427 Asn434 Tyr437 Gly438 Glu440 **Arg484 Ile487 Ala488 Arg491 Arg495**
Full-length protein in the open conformation (8REZ)		A	**Pro111 Val112 Ser113** Glu324 Glu325 Glu326 Thr330 Phe348 Gly349 Tyr350 Arg389 Phe390 Tyr391 Ser392 Pro393 Pro404 Val405 Asp406 Thr407 Tyr408 Ser409 Leu410 Arg414
Dimeric NBD extracted from the closed conformation (6R72)	1	A	Phe460 Glu463 Leu464 Pro465 Asp469 Thr470 Glu471 Gly476 Ile477 Met478 Leu479 Ser480 Gly481 Gly482 Gln483 Arg484 Ser508 Leu509 Asp510
	1	B	Tyr350 Lys351 Asp353 Gln354 Leu355 Ile356 Gly374 Pro375 Ser376 Gly377 Gly378 Gly379 Lys380 Thr381 Thr382 Lys385 Tyr391 Gln422 Glu423 Lys551
	2	A	Val420 Ser421 Gln422 Glu423 Ser424 Pro425 Leu426 Arg475 Gly476 Ile477 Gly481 Arg484 Gln485 Ala488, Ile489 Asp503 Ala504 Ala505 Ser507 Ser508
	2	B	Val420 Ser421 Gln422 Glu423 Ser424 Pro425 Leu426 Met427 Arg475 Ile477 Gly481 Arg484 Gln485 Ala488 Asp503 Ala504 Ala505 Ser507 Ser508
	3	B	Tyr350 Asp353 Gln354 Ile356 Pro375 Ser376 Gly377 Gly378 Gly379 Lys380 Thr381 Thr382 Lys385 Gln422 Glu423 Lys551
	3	A	Phe460 Glu463 Leu464 Pro465 Gly476 Ile477 Met478 Leu479 Ser480 Gly481 Gln483 Arg484
**Human ABCB1**			
Dimeric NBD from ABCB1 extracted from the closed conformation (6C0V)	1		Thr422 Leu552 Leu554 Gln556 Ala557 Thr558 Ser559 Ala560 Leu561 Asp562 Thr563 Glu566 Ala567 Gln570 Val571 Leu573 Asp574 Ala576 Arg577 Arg580 Thr582 Val584 Ile585 Ala586 His587 Arg588 Leu589 Ser590 Thr591 Val592 Arg593 Asn594 Ala595 His612 Tyr622 Val626 Met628 Gln629 Thr630 Thr1065 Leu1197 Leu1199 Asp1200 Gln1201 Ala1202 Thr1203 Ser1204 Ala1205 Leu1206 Asp1207 Thr1208 Glu1211 Lys1212 Gln1215 Leu1218 Asp1219 Arg1222 Arg1225 Cys1227 Val1229 Ile1230 Ala1231 His1232 Arg1233 Leu1234 Ser1235 Thr1236 Gln1238 Asn1239 Ala1240 Asp1241 Met1270 Val1273 Gln1274
	2		Glu476 Pro477 Val478 Leu479 Asp511 Phe512 Lys515 Leu516 Glu526 Arg527 Gly528 Ala529 Gln530 Leu531 Ser532 Gly533 Gln535 Lys536 Asp562 Thr563 Glu564 Asn1043 Tyr1044 Pro1045 Thr1046 Arg1047 Ile1050 Pro1051 Val1052 Val1069 Gly1070 Ser1071 Ser1072 Gly1073 Cys1074 Gly1075 Lys1076 Ser1077 Thr1078 Val1080 Gln1081 Glu1084 Phe1086 Tyr1087 Ile1115 Val1116 Ser1117 Gln1118 Glu1119

			Pro1120 Ile1121 K1172 Glu1200 Val1245 Phe1246 Gln1247 Asn1248 Gly1249 Ile1266 Ser1269 Met1270 Val1273
	3		Ser434 Val437 Gln438 Gln441 Leu443 Ile470 Gly471 Val472 Val473 Ser474 Gln475 Glu476 Pro477 Val478 Phe480 Tyr490 Gly491 Arg527 Ser532 Gly533 Lys536 Gln537 Ala540 Arg543 Ala544 Arg547 Asp555 Gln556 Ser559 Ala560 Gln1118 Glu1119 Pro1120 Asp1171 Lys1172 Gly1173 Thr1174 Leu1176 Ser1177 Gly1178 Lys1181 Gln1182 Gln1201 Ser1204 Ala1205
	4		Ser400 Tyr401 Pro402 Ser403 Arg404 Val407 Lys408 Ile409 Lys411 Gly427 Asn428 Ser429 Gly430 Cys431 Gly432 Lys433 Ser434 Thr435 Gln438 Tyr444 Phe601 Asp602 Asp603 Gly604 Ile621 Phe1157 Leu1161 Thr1174 Gln1175 Leu1176 Ser1177 Gln1180
	5		Glu1084 Phe1086 Gln1107 Arg1110 Ala1111 His1112 Leu1113 Gly1114 Ile1115 Val1116 Ser1117 Tyr1133 Gly1134 Asp1135 Asn1136 Ser1137 Arg1138 Ala1185 Arg1188 Ala1189 Arg1192
Full-length protein in the open conformation (8GMG)	1		ALA260 ILE261 ARG262 THR263 ILE265 ALA266 PHE267 VAL801 ILE1018 LEU1031 GLY1033 VAL1035 THR1036 PHE1037 VAL1040 PHE1042 LEU1053 GLY1055 LEU1056 SER1057 LEU1058 SER1077 VAL1079 VAL1080 GLN1081 LEU1082 LEU1083 GLU1084 ARG1085 PHE1086 TYR1087 ASP1088 PRO1089 VAL1094 LEU1095 LEU1096 ASP1097 ILE1101 LYS1102 LEU1104 ASN1105 VAL1106 LEU1109 ARG1110 ALA1111 ... ARG1192 PRO1194 HIS1195 ILE1196 ASP1200
	3		MET156 ARG157 GLN158 GLU159 ILE160 ILE375 SER377 SER434 VAL437 GLN438 MET440 GLN441 ARG442 LEU443 VAL463 ARG464 LEU466 ARG467 ILE470 GLY471 VAL472 VAL473 SER474 PRO477 VAL478 ALA540 ILE551 LEU552 LEU553 LEU554 ASP555 PHE904 ARG905 THR906 VAL908 SER909 THR911 GLU913
	4		PRO477 VAL478 LEU479 PHE480 ILE484 ALA485 ASN487 ILE488 TYR490 GLY491 MET497 ILE500 GLU501 ALA503 VAL504 ASN508 ALA509 TYR510 PHE512 ILE513 LEU516 PHE520 THR522 VAL524 GLY528 GLN530 LEU531 SER532 GLN535 LYS536 ARG538 ILE539 ALA540 ALA542 ARG543 GLU899 ALA900 ASN903 THR906 VAL907 LEU910 GLN912 PHE916
	6		LEU392 GLU393 PHE394 VAL397 PHE399 SER400 TYR401 ILE409 LEU410 LYS411 GLY412 LEU413 LEU415 LYS416 VAL417 LEU425 CYS431 GLY432 LYS433 THR435 THR436 VAL437 GLN438 LEU439 MET440 ARG442 TYR444 ASP445 PRO446 VAL451 SER452 VAL453 ILE458 ARG459 LEU553 LEU554 ASP555 ILE583 VAL584 ILE585 ALA599 GLY600 PHE601 GLY604 VAL605 ILE606
Monomer extracted from the open conformation (8GMG)	1		PRO477 VAL478 LEU479 PHE480 THR482 THR483 ILE484 ALA485 ASN487 ILE488 TYR490 MET497 ILE500 GLU501 ALA503 VAL504 ALA507 ASN508 ALA509 TYR510 PHE512 ILE513 LEU516 PRO517 HIS518 LYS519 PHE520 ASP521 THR522 LEU523 VAL524 GLY525 GLY528 ALA529 GLN530 LEU531 SER532 GLY534 GLN535 LYS536 GLN537 ARG538

			ILE539 ALA540 ALA542 ARG543 LEU561 SER565 GLU566 VAL568 VAL569
	2		PRO385 ASN387 ILE388 LYS389 GLY390 ASN391 LEU392 GLU393 PHE394 VAL397 PHE399 SER400 TYR401 ILE409 LEU410 LYS411 GLY412 LEU413 LEU415 LYS416 VAL417 LEU425 CYS431 GLY432 LYS433 THR435 THR436 VAL437 GLN438 LEU439 MET440 GLN441 ARG442 LEU443 TYR444 ASP445 PRO446 GLY449 MET450 VAL451 SER452 VAL453 ASP454 GLN456 ASP457 ILE458 ARG459 ILE461 PHE465 LEU466 ... LA599 GLY600 PHE601 GLY604 VAL605 ILE606
	3		GLY471 VAL472 VAL473 ALA503 GLU506 ALA507 ILE541 ALA542 ALA544 LEU545 ASN548 PRO549 ILE551 LEU552 LEU553 LEU554 ALA557 THR558 GLU566 ALA567 VAL569 GLN570 ALA572 LEU573 ALA576 ARG577 LYS578 GLY579 ARG580 THR582 ILE583 VAL584 ALA586 ARG588 SER590 THR591
	4		THR422 VAL423 ALA424 LEU425 VAL426 GLY427 ASN428 SER429 LYS433 SER434 ASP555 GLU556 GLN570 LEU573 ASP574 ARG577 THR582 ILE583 VAL584 ILE585 ALA586 HIS587 ARG588 LEU589 THR591 VAL592 ASN594 ALA595 LEU625 GLN629

In case of long sequences, continuous stretches of residues are indicated by "….."
